# Supplementary material for: Prebiotic magnetite enables chirality-magnetic surface feedback
Source: Sci Adv. 2026 Nov 6;12(45):eaeg3954. doi: 10.1126/sciadv.aeg3954 (PMC13742167; doi:10.1126/sciadv.aeg3954)
Supplement: Supplementary file 1 [file sciadv.aeg3954_sm.pdf]

Science Advances
AAAS

Supplementary Materials for

Prebiotic magnetite enables chirality-magnetic surface feedback

José A. P. M. Devienne *et al.*

Corresponding author: Furkan Oztur, ozturk@caltech.edu

Sci. Adv. **12**, eaeg3954 (2026)
DOI: 10.1126/sciadv.aeg3954

This PDF file includes:

Supplementary Text
Figs. S1 to S12
Tables S1 and S2
References

1 Introduction

In this Supplementary Information, we describe the procedures used to quantify the magnetite content of each synthesized sample and the supporting observations that informed these estimates. We first present additional electron microscopy data for the non-UV–synthesized materials, highlighting their overall morphology, phase associations, and textural variability. We then detail the image-processing workflow used to extract grain-scale information from these images. This includes the image segmentation strategy applied to isolate individual magnetite particles, the thresholding criteria adopted for phase discrimination, and the post-processing steps used to refine particle outlines. We report the resulting size and aspect-ratio distributions derived from the segmented images, which form the basis for the quantitative comparison discussed in the main text. We also include additional X-ray diffraction (XRD) data for the products of both the UV-driven and non-UV synthesis routes (Figure S4), where goethite peaks are more evident. Finally, high-temperature magnetic susceptibility measurements are included, where clear drop around 580 °C indicating the successful formation of magnetite in the synthetic samples.

2 Estimation of Magnetite Content

Magnetite abundances for all synthetic samples were determined by combining precise mass measurements with bulk magnetic characterization. Each powder was weighed before and after loading into the sample holder to obtain its net mass. The saturation magnetization (M_s) of the same powder was then measured from room-temperature hysteresis loops. Because magnetite has a well-established saturation magnetization of 92 Am^2kg^{-1} at room temperature (*34*), the mass of magnetite present in each sample was calculated by dividing the measured M_s (in Am^2) by this reference value. The resulting magnetite mass and its proportion relative to the total sample mass are reported in Table S1.

3 Image segmentation

As shown in the microscopy images (Figure S3 and S4), magnetite particles display a noticeable brightness contrast relative to the surrounding phases, allowing image segmentation and quantification of particle size and shape. Image processing was performed in ImageJ (*59*) following a standard workflow. First, each micrograph (e.g., Figure S5a) was converted to 8-bit grayscale to simplify the pixel intensity distribution. Contrast and brightness were then manually adjusted to enhance the distinction between magnetite grains and the surrounding matrix (Figure S5b). The optimized grayscale images were converted to binary (black–white) representations using a fixed threshold, isolating magnetite pixels as foreground objects. Finally, individual particles in the binary images were segmented and fitted using ellipses, from which equivalent diameters and aspect ratios were extracted (Figure S5c). The reconstructed size and shape distributions obtained from segmenting the microscopy images are shown in Figures S7-S10 and were used also in the Butler-Banerjee diagram plotted in Figure 3 in the main text.

4 Derivation of the exchange stiffness constant for an exchange-coupled system

Several studies of CISS-mediated interactions between chiral molecules and magnetic surfaces suggest that, when a spin-polarized molecule approaches or adsorbs onto a magnetic substrate, the molecular spin polarization can interact with spin-polarized surface electrons through an exchange-like interaction (*16*, *17*, *19*, *21*, *51*, *54*, *65*). At the microscopic level, this interaction can be written in a Heisenberg-like form,

$$E_{\text{pair}}^{\text{ex}} = -2J\,\mathbf{S}_{\text{mol}} \cdot \mathbf{S}_{\text{mag}}, \tag{S1}$$

where $\mathbf{S}_{\text{mol}}$ represents the spin polarization associated with the pole of the molecule in contact with the surface, $\mathbf{S}_{\text{mag}}$ represents a spin-polarized electron at the magnetite surface, and J is the exchange integral

describing the coupling between the two spins. For $J > 0$, parallel alignment is favored; for $J < 0$, antiparallel alignment is favored. For two spins with the same magnitude S, the energies of the parallel (P) and antiparallel (AP) configurations are

$$E_{\mathrm{P}} = -2JS^2, \tag{S2}$$

$$E_{\mathrm{AP}} = +2JS^2. \tag{S3}$$

Therefore, for $J > 0$, the energetic difference between antiparallel and parallel alignment is

$$\Delta E_{\mathrm{pair}} \equiv E_{\mathrm{AP}} - E_{\mathrm{P}} = 4JS^2. \tag{S4}$$

In the following, ΔE_{pair} is used as the magnitude of the per-interaction energy difference between the two spin configurations. The sign of the preferred alignment is encoded in the direction assigned to the molecular spin-polarization vector in the micromagnetic model. Thus, reversing the molecular handedness or reversing the preferred spin alignment is represented by replacing $\mathbf{p}$ with $-\mathbf{p}$.

If N molecules interact independently with spins at the magnetite surface, and if intermolecular interactions are neglected, the total additional exchange energy is

$$\begin{aligned} E_N^{\mathrm{ex}} &= -2J \sum_{n=1}^{N} \mathbf{S}_{\mathrm{mol},n} \cdot \mathbf{S}_{\mathrm{mag},n} \\ &\approx N E_{\mathrm{pair}}^{\mathrm{ex}}, \end{aligned} \tag{S5}$$

where the second line assumes that each molecule–surface interaction has the same exchange-energy scale. The corresponding antiparallel–parallel energy difference is then

$$\Delta E_N = N \Delta E_{\mathrm{pair}}. \tag{S6}$$

If the molecules cover an area A of the magnetic substrate, the molecular surface density is

$$n_s = \frac{N}{A}. \tag{S7}$$

Dividing Eq. (S6) by the coated surface area gives the antiparallel–parallel exchange-energy difference per unit area,

$$\Delta e_{\mathrm{mol}} = \frac{\Delta E_N}{A} = \frac{N}{A} \Delta E_{\mathrm{pair}} = n_s \Delta E_{\mathrm{pair}}. \tag{S8}$$

Eq. (S8) is the microscopic surface-energy bias produced by the adsorbed spin-polarized molecules. It depends only on the active molecular surface density n_s and the per-interaction exchange-energy difference ΔE_{pair}, both of which can be constrained from experiments involving adsorption of chiral molecules on magnetic substrates (*17*, *62*). In the next section, we derive the micromagnetic equivalent of this surface-energy bias and obtain the exchange stiffness constant assigned to the intermediate layer in the multi-phase micromagnetic model.

4.1 Micromagnetic exchange energy

In micromagnetics, the exchange energy of a magnetic body occupying a volume Ω is written in continuum form as

$$E_{\mathrm{exc}} = \int_{\Omega} A_{\mathrm{ex}} |\nabla \mathbf{m}|^2 \, d\Omega, \tag{S9}$$

where A_{ex} is the exchange stiffness constant and $\mathbf{m}(\mathbf{r})$ is the unit vector describing the local magnetization direction,

$$\mathbf{m}(x, y, z) = [m_x(x, y, z), m_y(x, y, z), m_z(x, y, z)] \,. \tag{S10}$$

The term $|\nabla \mathbf{m}|^2$ is the squared Frobenius norm of the magnetization gradient tensor,

$$|\nabla \mathbf{m}|^2 = \sum_{i\in\{x,y,z\}} \sum_{j\in\{x,y,z\}} \left(\frac{\partial m_i}{\partial x_j}\right)^2 . \tag{S11}$$

In our multiphase model, the system is divided into three blocks: Block 1 represents the magnetite particle and has the micromagnetic parameters of magnetite, namely saturation magnetization M_s, magnetocrystalline anisotropy K_1, and exchange stiffness A_{ex}; block 2 represents a thin intermediate layer (IL); and block 3 represents the spin-polarized molecular layer. For blocks 2 and 3, we set $M_s = 0$ and $K_1 = 0$, so these blocks do not contribute magnetostatic or anisotropy energy. The molecular layer is pinned along a fixed unit vector $\mathbf{p}$, representing the molecular spin-polarization direction. The intermediate layer is assigned a nonzero exchange stiffness $A_{\mathrm{ex}}^{\mathrm{IL}}$, allowing it to mediate an exchange-like coupling between the magnetite surface and the pinned molecular layer. The goal of this derivation is to determine the value of $A_{\mathrm{ex}}^{\mathrm{IL}}$ required for the continuum micromagnetic model to reproduce the molecular surface-energy bias given by Eq. (S8).

We consider a magnetite particle with a coated surface area S, separated from the pinned molecular layer by an intermediate layer of thickness d. The volume of the intermediate layer is therefore

$$\Omega_{\mathrm{IL}} = Sd. \tag{S12}$$

The exchange energy stored in the intermediate layer is

$$\begin{aligned} E_{\mathrm{ex}}^{\mathrm{IL}} &= \int_{\Omega_{\mathrm{IL}}} A_{\mathrm{ex}}^{\mathrm{IL}} |\nabla \mathbf{m}_{\mathrm{IL}}|^2 \, d\Omega \\ &= \int_S \int_0^d A_{\mathrm{ex}}^{\mathrm{IL}} |\nabla \mathbf{m}_{\mathrm{IL}}|^2 \, dz \, dS, \end{aligned} \tag{S13}$$

where $\mathbf{m}_{\mathrm{IL}}$ is the magnetization-direction field within the intermediate layer. By construction, $A_{\mathrm{ex}}^{\mathrm{IL}}$ is uniform within this layer.

The intermediate layer is deliberately chosen to be very thin compared with the lateral dimensions of the coated magnetite surface. Its lower boundary is set by the magnetite surface magnetization,

$$\mathbf{m}_{\mathrm{IL}}(x, y, 0) = \mathbf{m}_0(x, y), \tag{S14}$$

while its upper boundary is fixed by the spin-polarized molecular layer,

$$\mathbf{m}_{\mathrm{IL}}(x, y, d) = \mathbf{p}. \tag{S15}$$

Because the two boundary conditions are imposed across a small thickness d, the dominant variation of $\mathbf{m}_{\mathrm{IL}}$ occurs in the direction normal to the interface, here chosen as z. In contrast, variations along x and y occur over the lateral length scale of the magnetite surface state, which is typically much larger than d. Therefore, to leading order,

$$\left|\frac{\partial m_i^{\mathrm{IL}}}{\partial x}\right| \ll \left|\frac{\partial m_i^{\mathrm{IL}}}{\partial z}\right|, \qquad \left|\frac{\partial m_i^{\mathrm{IL}}}{\partial y}\right| \ll \left|\frac{\partial m_i^{\mathrm{IL}}}{\partial z}\right|, \qquad i \in \{x, y, z\}. \tag{S16}$$

Under this thin-layer approximation,

$$|\nabla \mathbf{m}_{\mathrm{IL}}|^2 \approx \left|\frac{\partial \mathbf{m}_{\mathrm{IL}}}{\partial z}\right|^2 . \tag{S17}$$

The derivative across the intermediate layer can then be approximated by a finite difference between the two boundary values,

$$\frac{\partial \mathbf{m}_{\mathrm{IL}}}{\partial z} \approx \frac{\mathbf{m}_{\mathrm{IL}}(x,y,d) - \mathbf{m}_{\mathrm{IL}}(x,y,0)}{d} = \frac{\mathbf{p} - \mathbf{m}_0(x,y)}{d}. \tag{S18}$$

Substituting Eqs. (S17) and (S18) into Eq. (S13) gives

$$\begin{aligned} E_{\mathrm{ex}}^{\mathrm{IL}} &\approx \int_S \int_0^d A_{\mathrm{ex}}^{\mathrm{IL}} \left| \frac{\mathbf{p} - \mathbf{m}_0(x,y)}{d} \right|^2 dz\, dS \\ &= \frac{S A_{\mathrm{ex}}^{\mathrm{IL}}}{d^2} \int_0^d |\mathbf{p} - \mathbf{m}_0(x,y)|^2\, dz. \end{aligned} \tag{S19}$$

Because both $\mathbf{p}$ and $\mathbf{m}_0$ are unit vectors,

$$\begin{aligned} |\mathbf{p} - \mathbf{m}_0|^2 &= (\mathbf{p} - \mathbf{m}_0) \cdot (\mathbf{p} - \mathbf{m}_0) \\ &= |\mathbf{p}|^2 + |\mathbf{m}_0|^2 - 2\mathbf{p} \cdot \mathbf{m}_0 \\ &= 2\left(1 - \mathbf{p} \cdot \mathbf{m}_0\right). \end{aligned} \tag{S20}$$

Therefore, the exchange energy stored in the intermediate layer can be written in surface form as

$$E_{\mathrm{ex}}^{\mathrm{IL}} \approx \frac{2 S A_{\mathrm{ex}}^{\mathrm{IL}}}{d^2} \int_z \left[1 - \mathbf{p} \cdot \mathbf{m}_0(x,y)\right] dz. \tag{S21}$$

For a locally uniform surface magnetization, this gives the interfacial exchange-energy density

$$e_{\mathrm{int}} = \frac{E_{\mathrm{ex}}^{\mathrm{IL}}}{S} \approx \frac{2 A_{\mathrm{ex}}^{\mathrm{IL}}}{d} \left(1 - \mathbf{p} \cdot \mathbf{m}_0\right). \tag{S22}$$

Eq. (S22) is the continuum micromagnetic equivalent of an exchange-like interfacial coupling between the magnetite surface magnetization and the fixed molecular spin-polarization direction. The energy is minimized when $\mathbf{m}_0$ is parallel to $\mathbf{p}$ and maximized when $\mathbf{m}_0$ is antiparallel to $\mathbf{p}$. Therefore,

$$e_{\mathrm{P}} = \frac{2 A_{\mathrm{ex}}^{\mathrm{IL}}}{d} \left(1 - \mathbf{p} \cdot \mathbf{p}\right) = 0, \tag{S23}$$

$$e_{\mathrm{AP}} = \frac{2 A_{\mathrm{ex}}^{\mathrm{IL}}}{d} \left[1 - \mathbf{p} \cdot (-\mathbf{p})\right] = \frac{4 A_{\mathrm{ex}}^{\mathrm{IL}}}{d}. \tag{S24}$$

The antiparallel–parallel energy difference per unit area generated by the micromagnetic intermediate layer is therefore

$$\Delta e_{\mathrm{int}} \equiv e_{\mathrm{AP}} - e_{\mathrm{P}} = \frac{4 A_{\mathrm{ex}}^{\mathrm{IL}}}{d}. \tag{S25}$$

On the microscopic side, Eq. (S8) gives the antiparallel–parallel energy difference per unit area produced by the adsorbed molecules. To make the continuum micromagnetic model reproduce the same areal energetic bias as the molecular spin-exchange interaction, we equate these two surface-energy differences,

$$\frac{4 A_{\mathrm{ex}}^{\mathrm{IL}}}{d} = n_s \Delta E_{\mathrm{pair}}. \tag{S26}$$

Solving for the exchange stiffness assigned to the intermediate layer yields

$$A_{\mathrm{ex}}^{\mathrm{IL}} = \frac{n_s \Delta E_{\mathrm{pair}}\, d}{4}. \tag{S27}$$

Eq. (S27) is the expression used in the micromagnetic simulations. It maps the molecular-scale exchange preference, expressed through ΔE_{pair} and the active surface density n_s, onto the continuum exchange stiffness required for the intermediate layer. The thickness d is therefore not interpreted as the physical molecule–surface interaction distance. Instead, d is the numerical thickness over which the interfacial energy is distributed in the finite-element mesh. When $A_{\mathrm{ex}}^{\mathrm{IL}}$ is rescaled according to Eq. (S27), the physically relevant quantity remains the surface energetic bias $n_s \Delta E_{\mathrm{pair}}$.

4.2 Model validation of the interfacial exchange mapping

To validate Eq. (S27) and ensure that the mapped A_{exc}^{IL}reproduces the intended additional exchange energy imposed by the molecular layer, we performed a series of simulations for different intermediate-layer thicknesses d and target interfacial energy differences ΔE_{exc}^{mol}. For each pair (d, ΔE_{exc}^{mol}), we computed the micromagnetic energy difference between antiparallel and parallel uniform end states in magnetite cubic particles of different size and compared the recovered ΔE_{exc}^{mic} with the theoretical target value (Figure S12).

As discussed above, the mapping assumes that the dominant magnetization variation within the IL occurs along the pinned molecular polarization axis **p**, and that the IL thickness remains small compared to the particle dimensions. These assumptions are best satisfied for thin layers and for particles large compared to the IL thickness. Figure S12 shows that, for the smallest grains, increasing d progressively overestimates the micromagnetic exchange-energy difference because the thicker IL introduces additional exchange-penalized rotation. As particle size increases, the discrepancy becomes negligible and the micromagnetic and theoretical energy differences converge. On this basis, a thin intermediate layer of thickness $d = 1$ nm was adopted in the main calculations so that the imposed interfacial bias remains close to the intended ΔE_{exc} over the size range of interest.

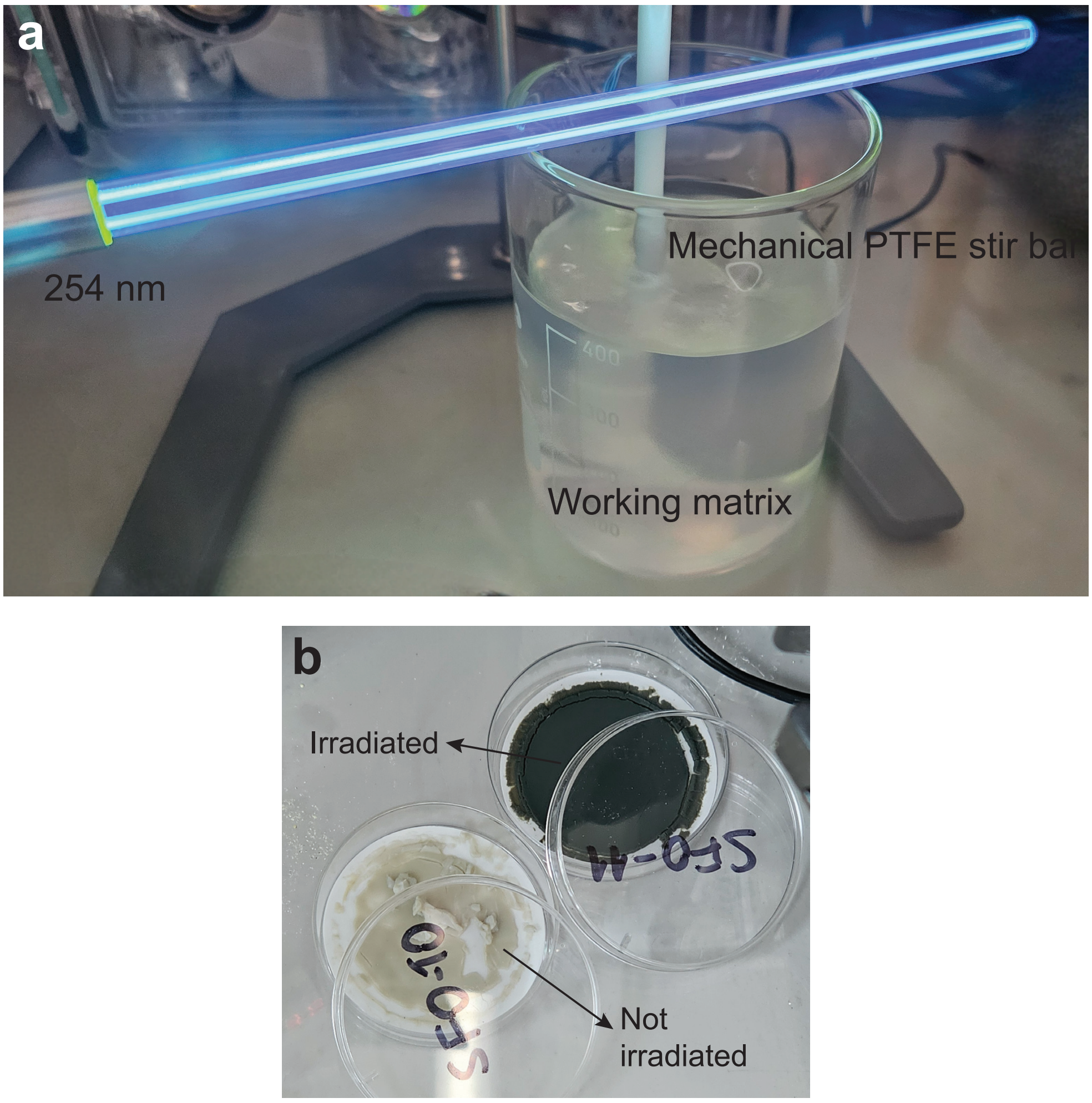

Figure S1. **a** Image from the irradiation experiments showing the experimental apparatus placed inside an anoxic glove box. **b** Irradiated and non-irradiated samples immediately after sediment collection. The irradiated samples exhibit a greenish-gray appearance due to the presence of green rust and iron oxides, whereas the non-irradiated samples appear white, as they predominantly contain iron carbonates and white rust.

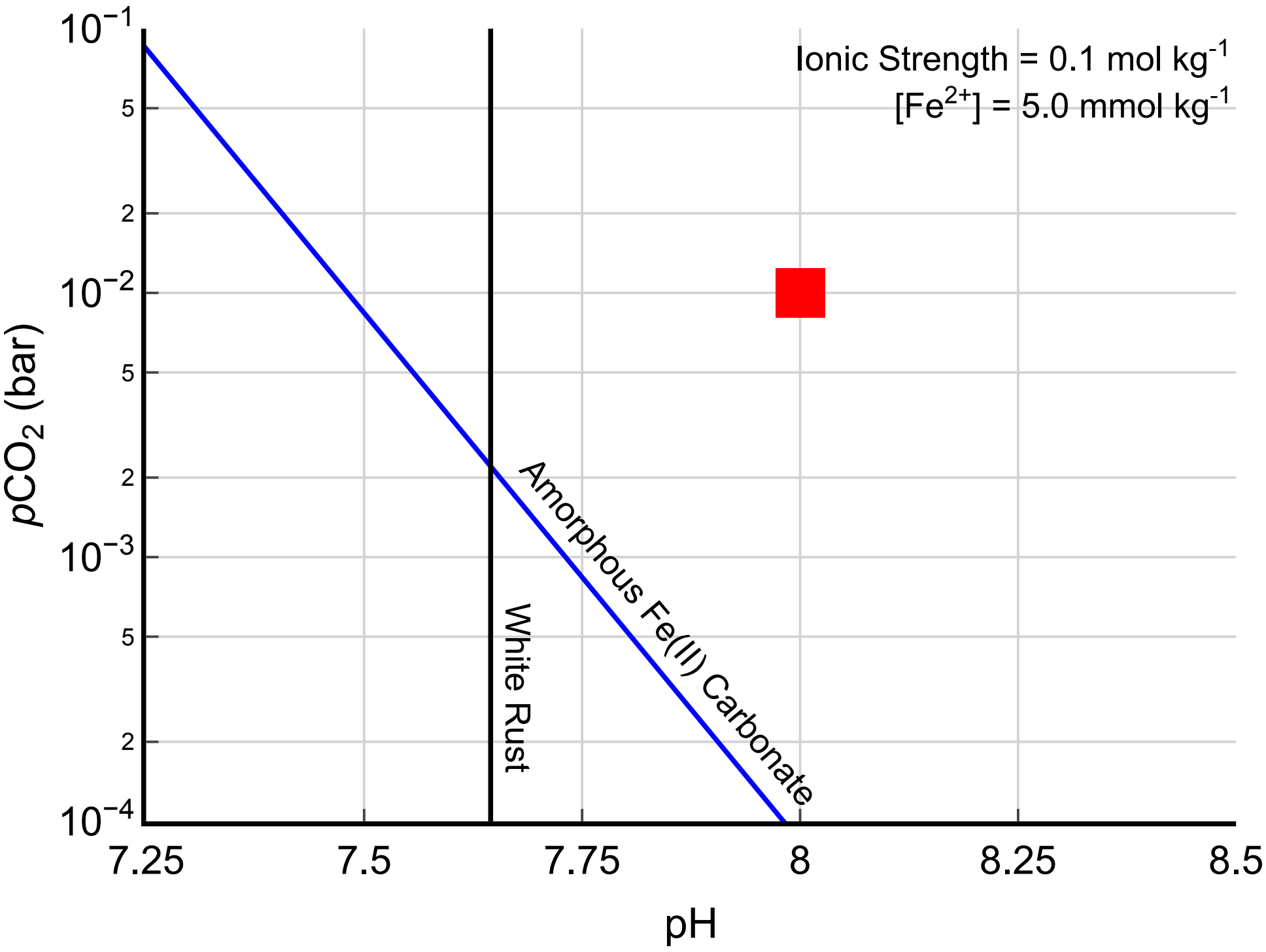

Figure S2. Thermodynamic phase diagram of pCO_2 versus pH, showing the corresponding solubility lines for amorphous ferrous iron carbonate and white rust. The red dot indicates the selected parameter conditions used for preparation of the working matrix in the magnetite synthesis experiments.

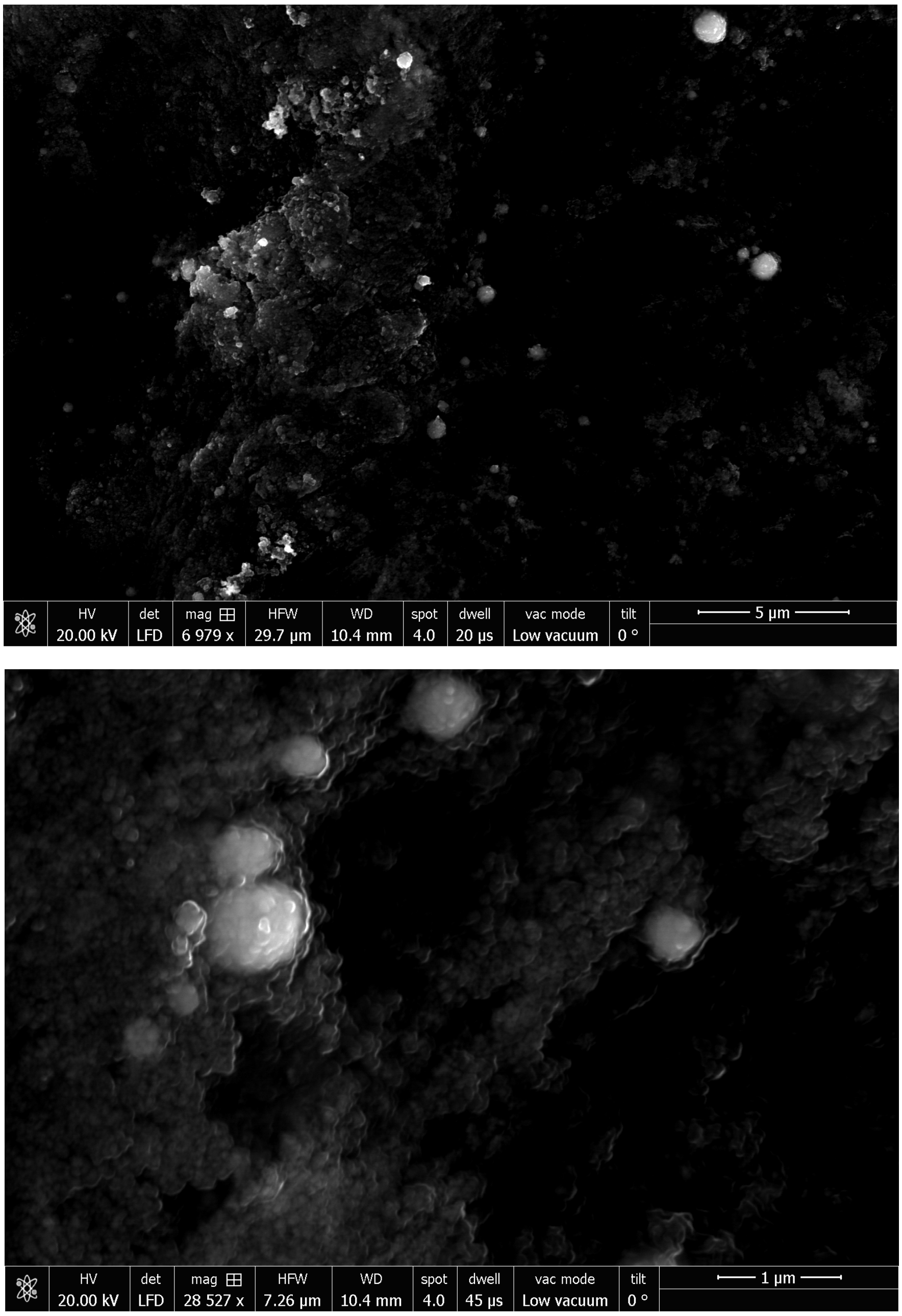

Figure S3. Electron microscopy imaging of sample NUV6.

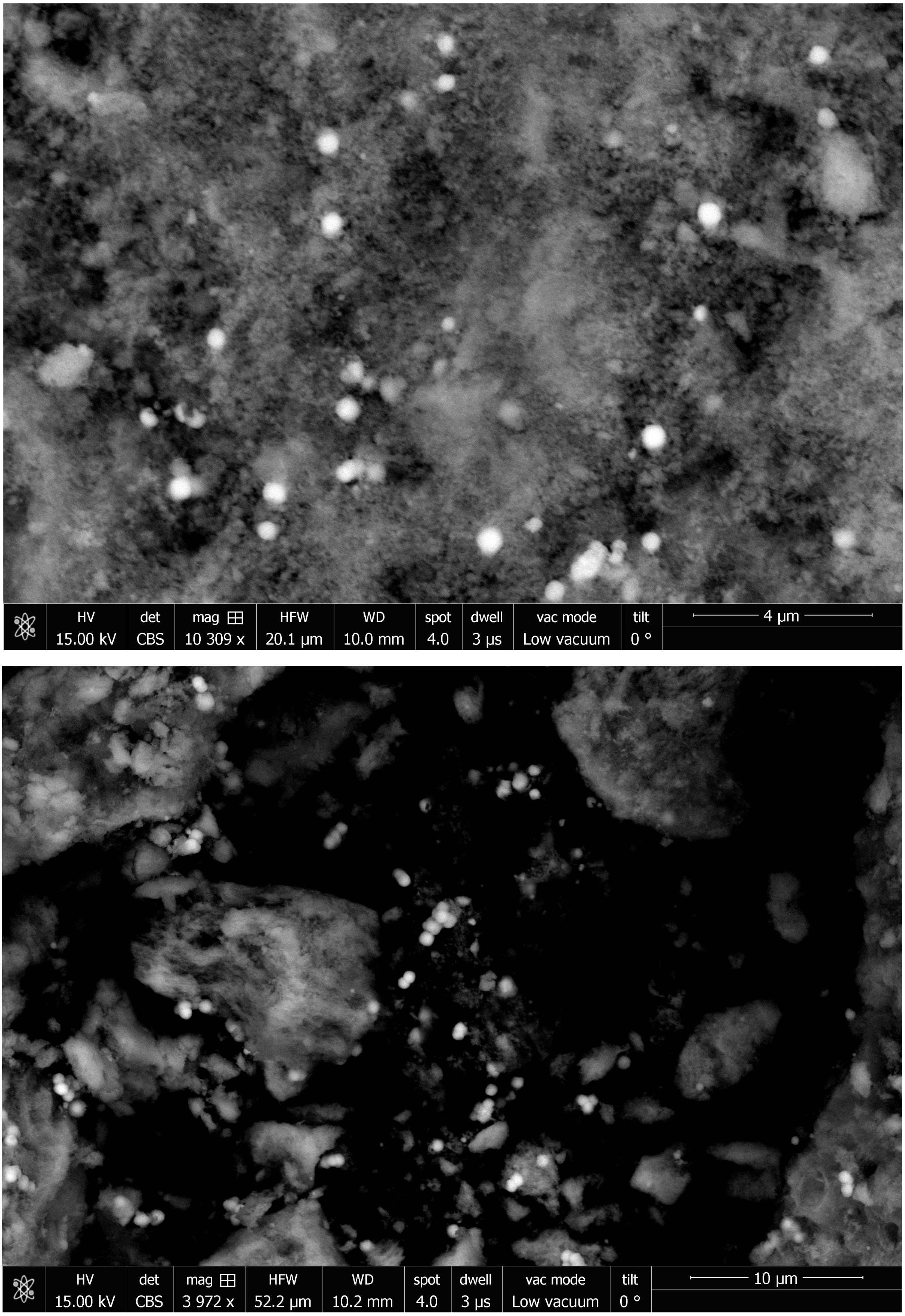

Figure S4. Electron microscopy imaging of sample NUV8 (top) and NUV9 (bottom).

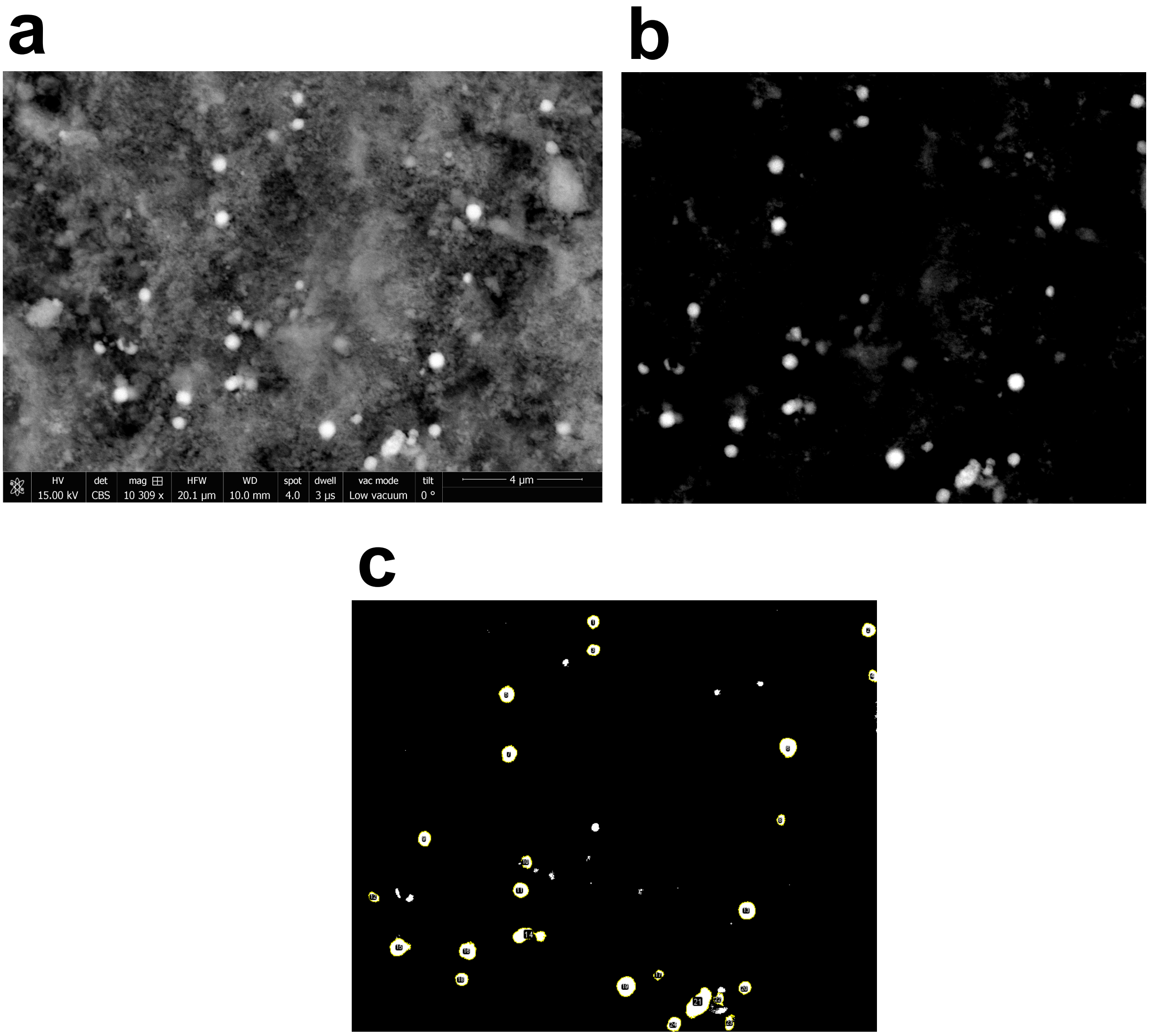

Figure S5. Particle size and shape reconstruction from sample NUV8 (same as shown in Figure S4) using image segmentation with ImageJ.

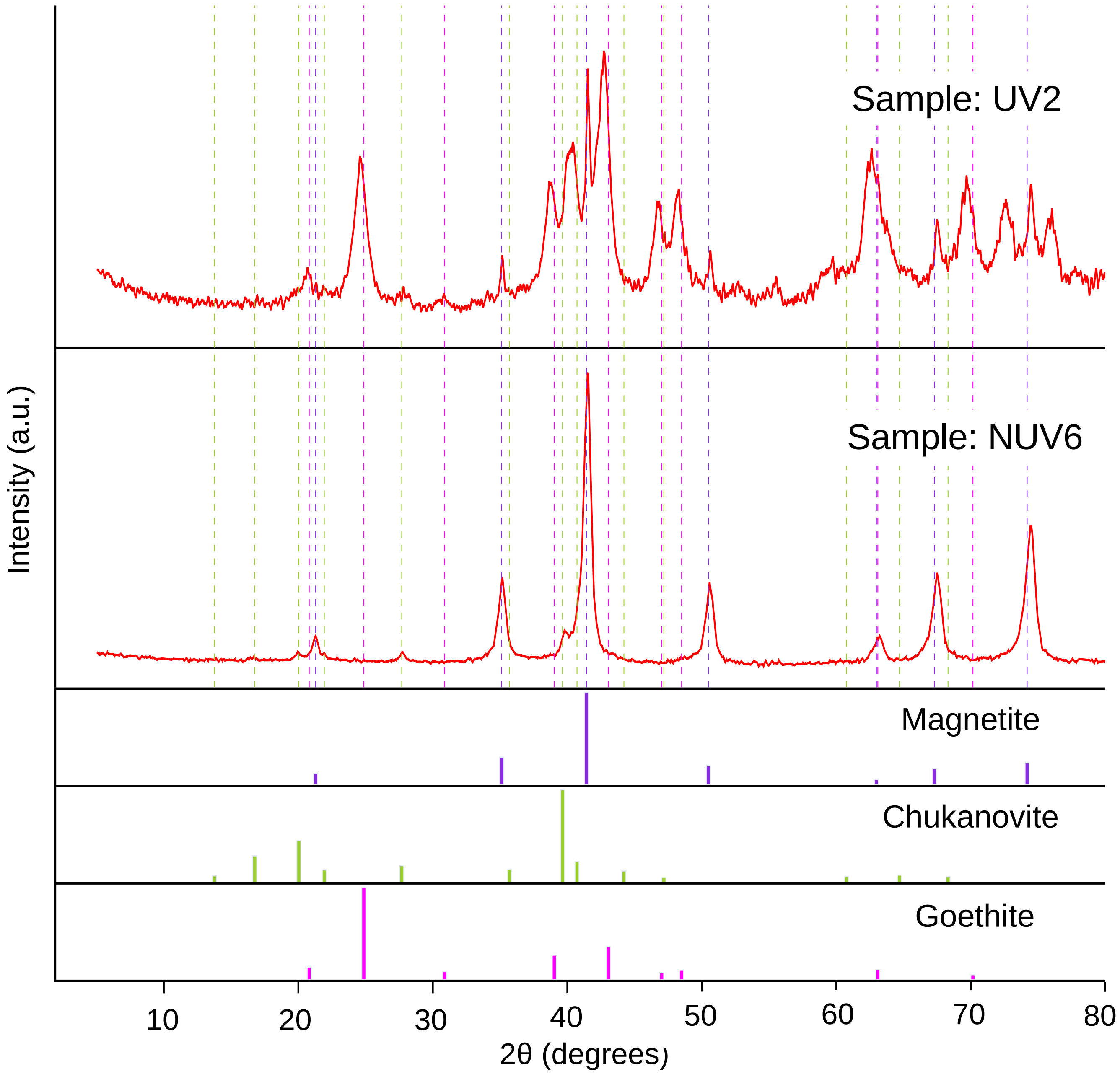

Figure S6. X-ray diffraction of sample UV2 and NUV6.

Table S1: Magnetite content and magnetic hysteresis parameters of synthetic samples. Saturation and remanent magnetizations (M_s and M_r) are in μA·m^2; B_c and B_{cr} are in mT.

**Sample**	**Magnetite mass** (μg)	**Magnetite percentage** (%)	**Saturation magnetization, M_s** (μA·m^2)	**Remanent magnetization, M_{rs}** (μA·m^2)	**Coercivity B_c** (mT)	**Remanence coercivity, B_{cr}** (mT)	**M_r/M_s**	**B_{cr}/B_c**
UV1	4.01	1.34	0.471 316	0.021 348	4.554	24.411	0.045	5.360
UV2	3.19	0.36	0.421 208	0.026 018	5.190	21.573	0.062	4.157
UV3	108.52	7.92	0.306 848	0.069 376	8.367	18.750	0.226	2.241
NUV5	5.53	1.97	0.349 908	0.052 965	5.677	15.331	0.151	2.701
NUV6	378.53	42.06	16.073 000	0.977 778	2.122	7.006	0.061	3.301
NUV7	77.85	7.41	5.489 480	0.991 032	7.774	18.343	0.181	2.360
NUV8	128.06	16.85	0.107 050	0.008 901	5.599	15.699	0.083	2.804
NUV9	5.53	7.90	0.228 669	0.025 817	4.693	16.612	0.113	3.540

Table S2: VARIFORC optimization parameters used for each sample.

Sample	S_{c0}	S_{Sc1}	S_{b0}	S_{b1}	λ_h	λ_v
UV1	9	9	4	9	0.2	0.2
UV2	5	9	4	9	0.2	0.2
UV3	5	9	4	9	0.2	0.2
NUV5	7	7	6	7	0.2	0.2
NUV6	5	8	4	8	0.1	0.1
NUV7	7	9	8	6	0.2	0.2
NUV8	5	9	5	9	0.2	0.2
NUV9	6	9	6	9	0.2	0.2

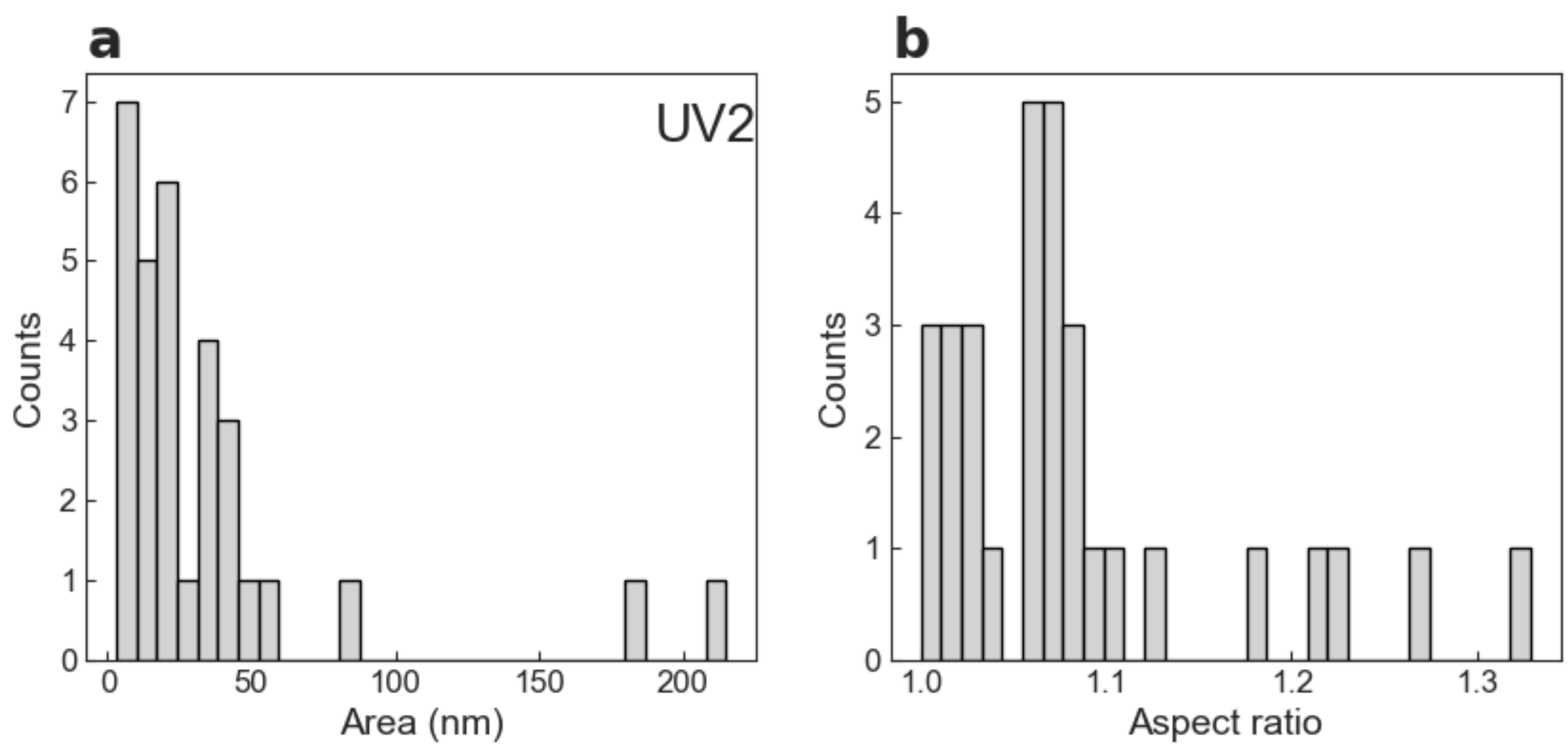

Figure S7. Size and shape distribution reconstructed from image segmentation of electron microscopy imaging of sample UV2 (UV-driven synthesis).

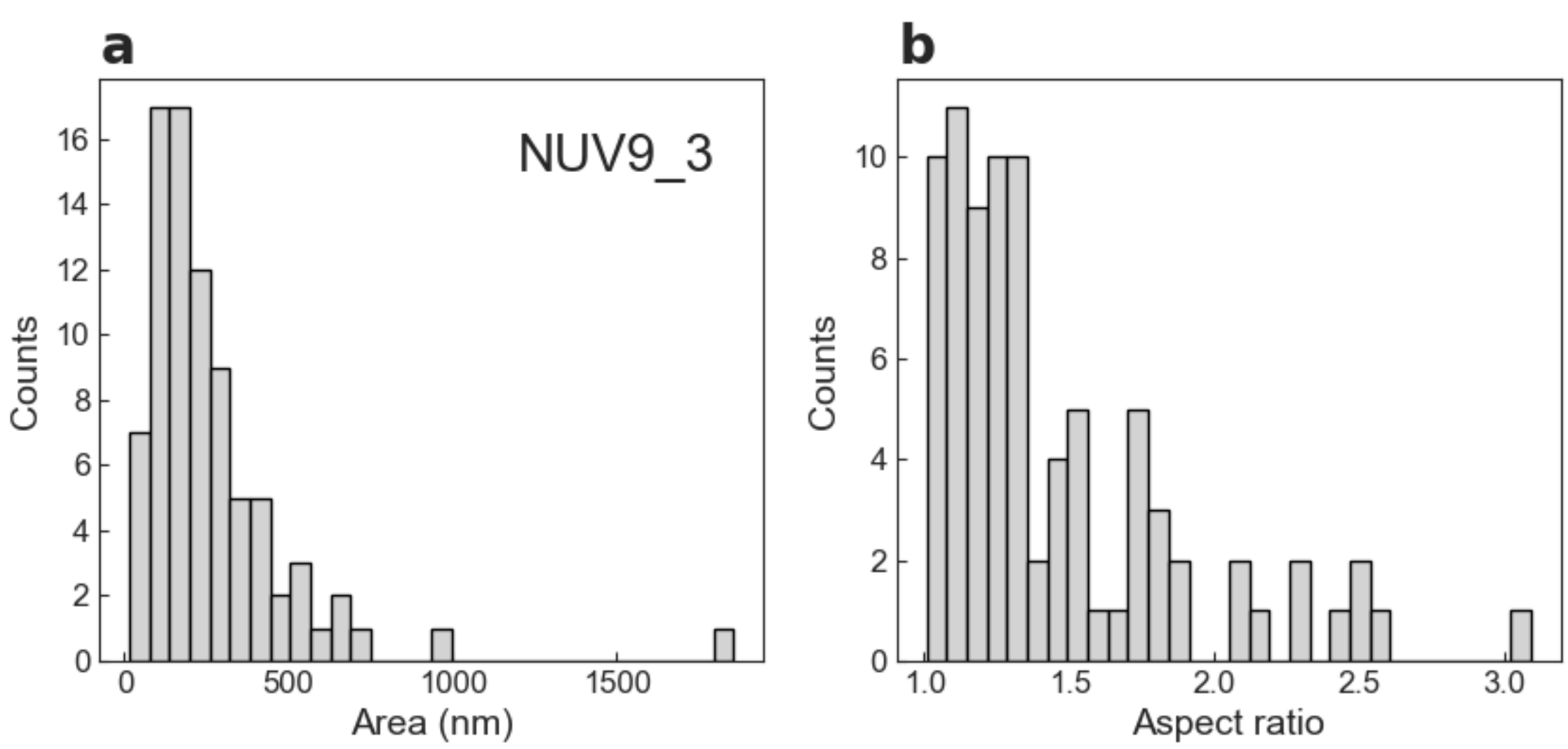

Figure S8. Size and shape distribution reconstructed from image segmentation of electron microscopy imaging of sample NUV9 (NO_2^--driven synthesis). NO_2^- concentration is 20 μM.

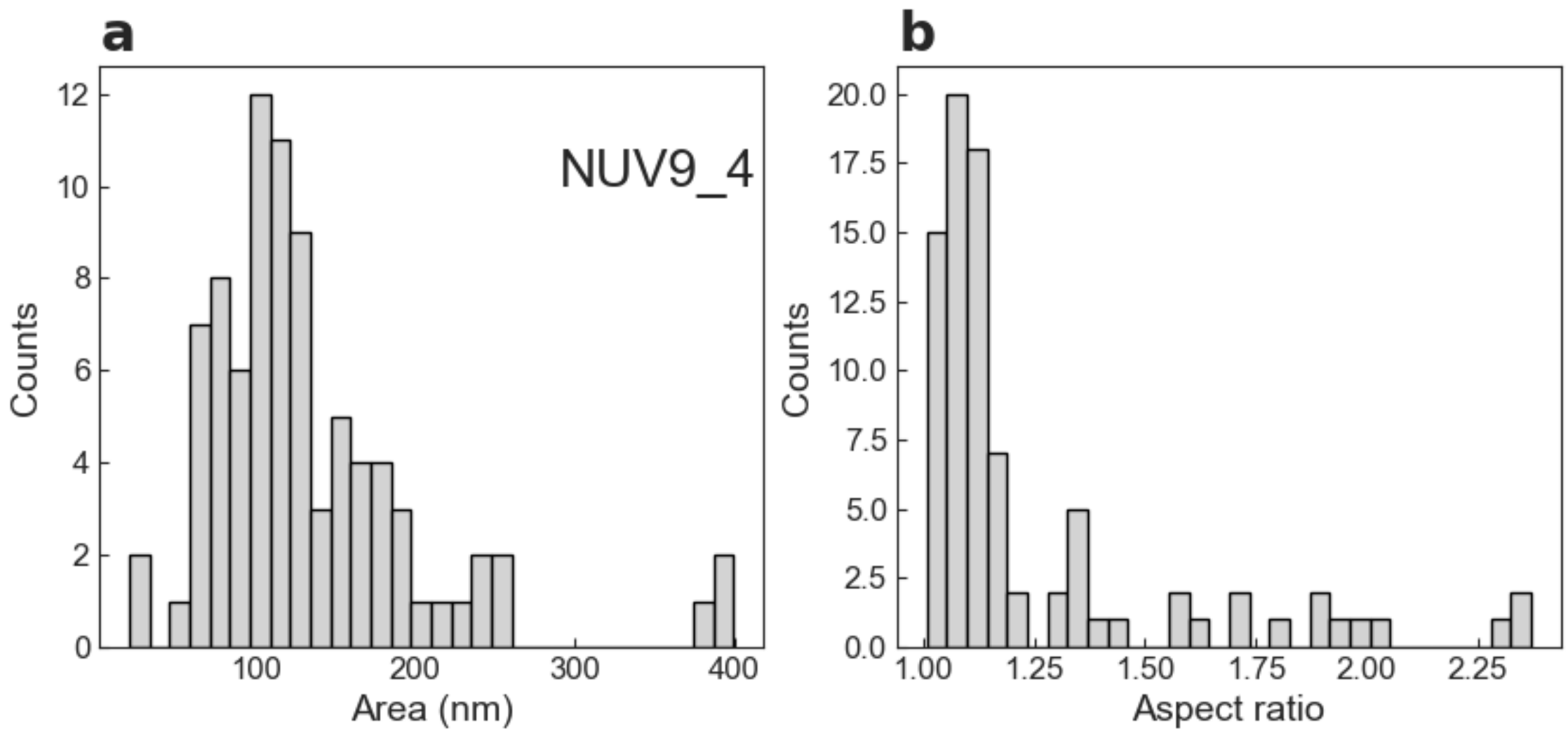

Figure S9. Size and shape distribution reconstructed from image segmentation of electron microscopy imaging of sample NUV9 (NO_2^--driven synthesis). NO_2^- concentration is 100 μM.

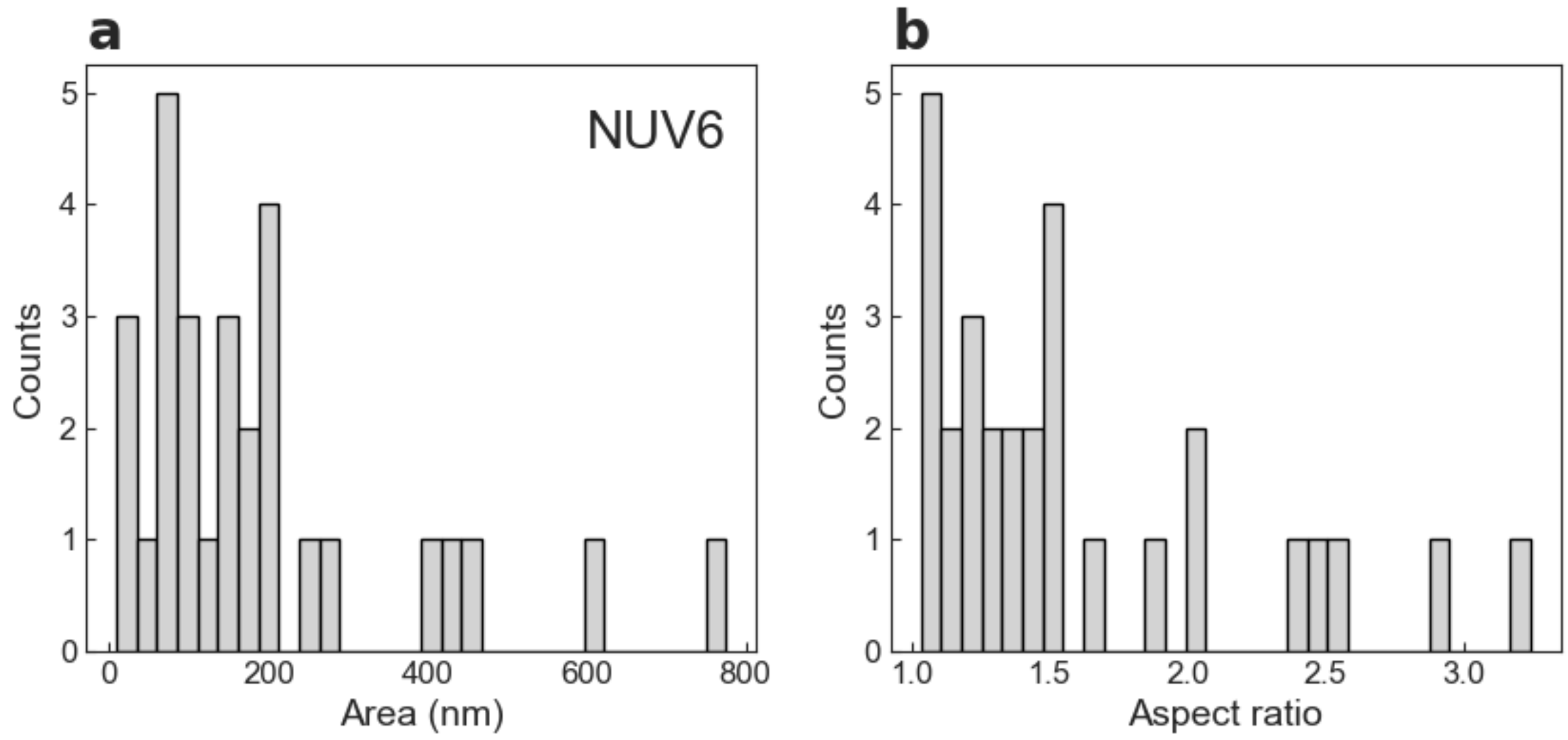

Figure S10. Size and shape distribution reconstructed from image segmentation of electron microscopy imaging of sample NUV6 (Fe^{II}/Fe^{III}-mixture driven synthesis).

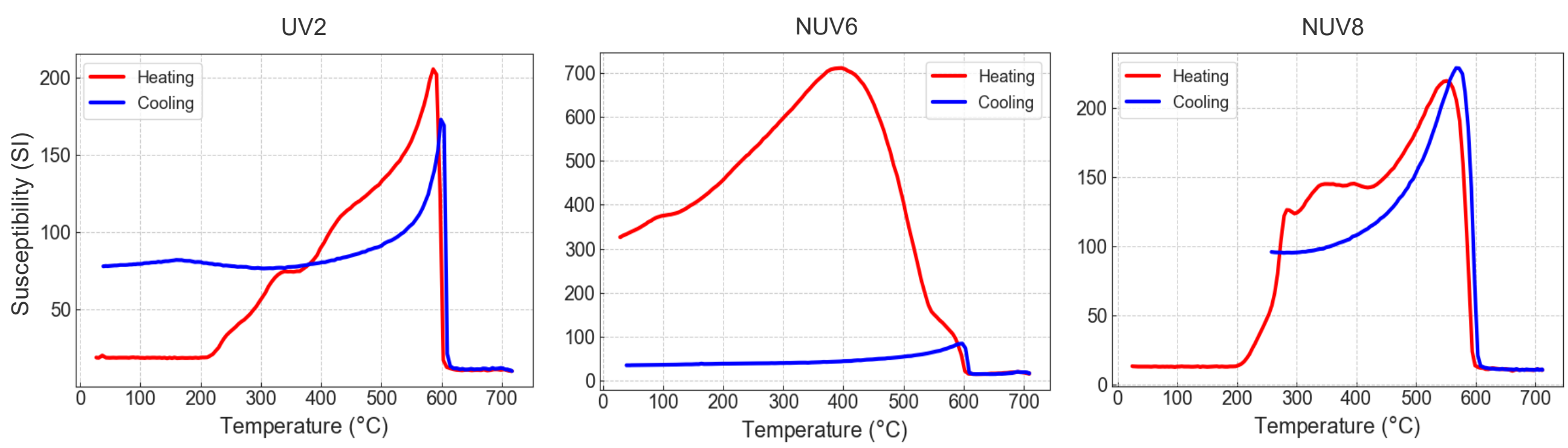

Figure S11. Examples of high-temperature magnetic susceptibility measurements of three representative samples: UV2 (UV-driven synthesis, left), NUV6 (Fe(II)/Fe(III) mixture synthesis, center) and NUV8 (NO_2^--driven, right). In red (blue) is the heating (cooling) curve.

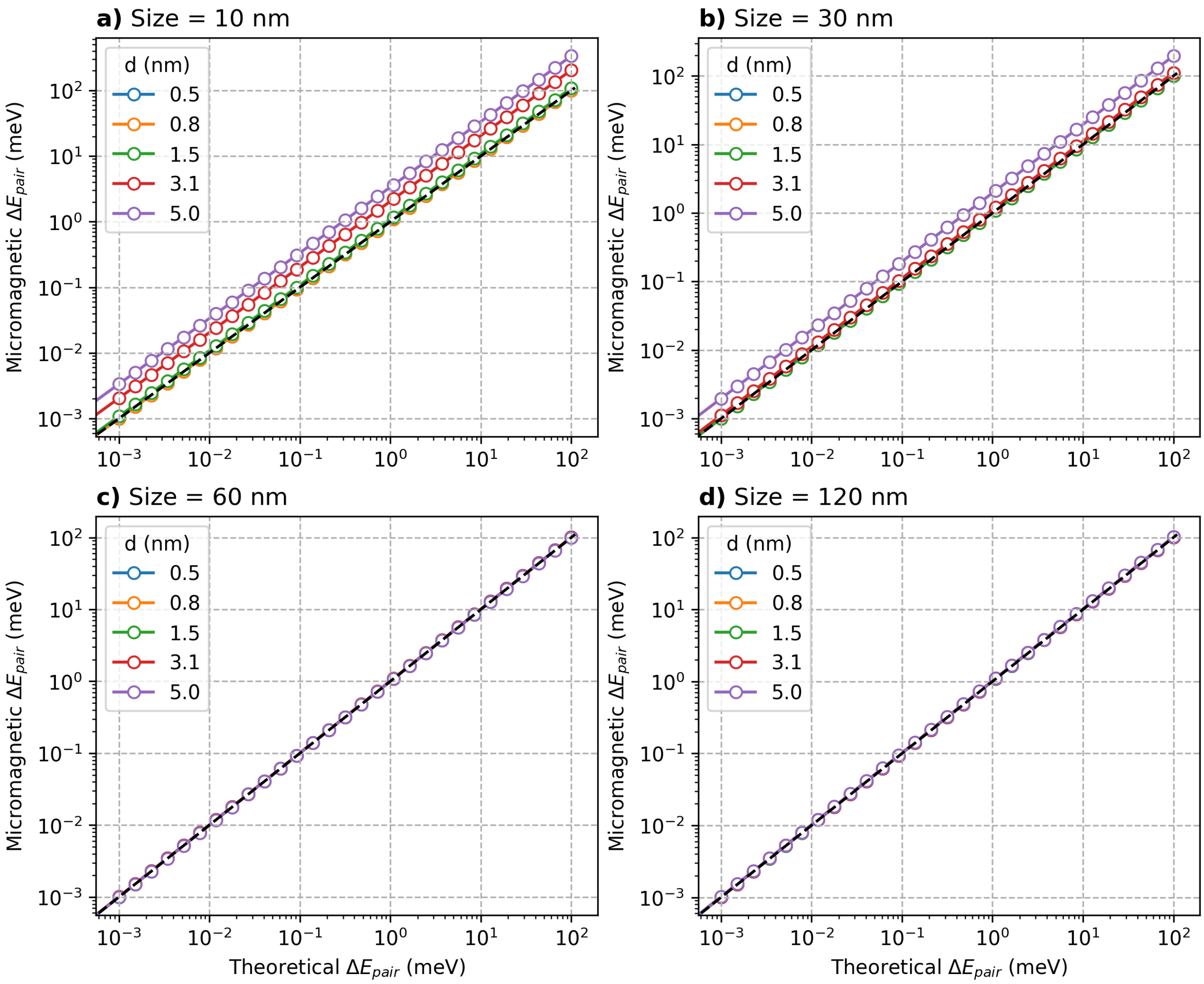

Figure S12. Numerical validation of the interfacial exchange-energy mapping used in Eq. 5 in the main manuscript. Comparison between the prescribed theoretical exchange-energy difference, ΔE_{pair}, and the additional micromagnetic exchange-energy contribution generated by the multiphase implementation for particles of different sizes and intermediate-layer thicknesses d. The dashed line shows the expected one-to-one relation. For the particle sizes and thin interfacial layers used in the manuscript, the micromagnetic energy contribution reproduces the prescribed ΔE_{pair}, confirming that Eq. 5 correctly maps the molecule-scale exchange preference into the coarse-grained micromagnetic model. Deviations occur for very small particles and artificially large d, where the assumption of a sharp magnetization change across the intermediate layer becomes less accurate and the coupling is overestimated.

REFERENCES

1. D. G. Blackmond, The origin of biological homochirality. *Cold Spring Harb. Perspect. Biol.* **11**, a032540 (2019).

2. Q. Sallembien, L. Bouteiller, J. Crassous, M. Raynal, Possible chemical and physical scenarios towards biological homochirality. *Chem. Soc. Rev.* **51**, 3436–3476 (2022).

3. S. F. Ozturk, D. D. Sasselov, On the origins of life's homochirality: Inducing enantiomeric excess with spin-polarized electrons. *Proc. Natl. Acad. Sci. U.S.A.* **119**, e2204765119 (2022).

4. S. F. Ozturk, D. D. Sasselov, J. D. Sutherland, The central dogma of biological homochirality: How does chiral information propagate in a prebiotic network? *J. Chem. Phys.* **159**, 061102 (2023).

5. S. F. Ozturk, thesis, Harvard University Graduate School of Arts and Sciences (2024).

6. S. F. Ozturk, D. D. Sasselov, Life's homochirality: Across a prebiotic network. *Proc. Natl. Acad. Sci. U.S.A.* **122**, e2505126122 (2025).

7. D. D. Sasselov, J. P. Grotzinger, J. D. Sutherland, The origin of life as a planetary phenomenon. *Sci. Adv.* **6**, eaax3419 (2020).

8. D. K. Kondepudi, G. W. Nelson, Weak neutral currents and the origin of biomolecular chirality. *Nature* **314**, 438–441 (1985).

9. J. Bailey, A. Chrysostomou, J. Hough, T. Gledhill, A. McCall, S. Clark, F. Menard, M. Tamura, Circular polarization in star-formation regions: Implications for biomolecular homochirality. *Science* **281**, 672–674 (1998).

10. C. Viedma, Chiral symmetry breaking during crystallization: Complete chiral purity induced by nonlinear autocatalysis and recycling. *Phys. Rev. Lett.* **94**, 065504 (2005).

11. R. Rosenberg, M. A. Haija, P. Ryan, Chiral-selective chemistry induced by spin-polarized secondary electrons from a magnetic substrate. *Phys. Rev. Lett.* **101**, 178301 (2008).

12. W. L. Noorduin, A. A. Bode, M. Van Der Meijden, H. Meekes, A. F. Van Etteger, W. J. Van Enckevort, P. C. Christianen, B. Kaptein, R. M. Kellogg, T. Rasing, E. Vlieg, Complete chiral symmetry breaking of an amino acid derivative directed by circularly polarized light. *Nat. Chem.* **1**, 729–732 (2009).

13. N. Globus, R. D. Blandford, The chiral puzzle of life. *Astrophys. J. Lett.* **895**, L11 (2020).

14. C. Meinert, P. De Marcellus, L. L. S. dHendecourt, L. Nahon, N. C. Jones, S. V. Hoffmann, J. H. Bredehoft, U. J. Meierhenrich, Photochirogenesis: Photochemical models on the absolute asymmetric formation of amino acids in interstellar space. *Phys. Life Rev.* **8**, 307–330 (2011).

15. D. G. Blackmond, Autocatalytic models for the origin of biological homochirality. *Chem. Rev.* **120**, 4831–4847 (2019).

16. S. F. Ozturk, Z. Liu, J. D. Sutherland, D. D. Sasselov, Origin of biological homochirality by crystallization of an RNA precursor on a magnetic surface. *Sci. Adv.* **9**, eadg8274 (2023).

17. O. Ben Dor, S. Yochelis, A. Radko, K. Vankayala, E. Capua, A. Capua, S.-H. Yang, L. T. Baczewski, S. S. P. Parkin, R. Naaman, Y. Paltiel, Magnetization switching in ferromagnets by adsorbed chiral molecules without current or external magnetic field. *Nat. Commun.* **8**, 14567 (2017).

18. K. Banerjee-Ghosh, O. B. Dor, F. Tassinari, E. Capua, S. Yochelis, A. Capua, S.-H. Yang, S. S. P. Parkin, S. Sarkar, L. Kronik, L. T. Baczewski, R. Naaman, Y. Paltiel, Separation of enantiomers by their enantiospecific interaction with achiral magnetic substrates. *Science* **360**, 1331–1334 (2018).

19. G. Koplovitz, G. Leitus, S. Ghosh, B. P. Bloom, S. Yochelis, D. Rotem, F. Vischio, M. Striccoli, E. Fanizza, R. Naaman, D. H. Waldeck, D. Porath, Y. Paltiel, Single domain 10 nm ferromagnetism imprinted on superparamagnetic nanoparticles using chiral molecules. *Small* **15**, e1804557 (2019).

20. B. P. Bloom, Y. Paltiel, R. Naaman, D. H. Waldeck, Chiral induced spin selectivity. *Chem. Rev.* **124**, 1950–1991 (2024).

21. S. F. Ozturk, D. K. Bhowmick, Y. Kapon, Y. Sang, A. Kumar, Y. Paltiel, R. Naaman, D. D. Sasselov, Chirality-induced avalanche magnetization of magnetite by an RNA precursor. *Nat. Commun.* **14**, 6351 (2023).

22. L.-F. Wu, M. Su, Z. Liu, S. J. Bjork, J. D. Sutherland, Interstrand aminoacyl transfer in a tRNA acceptor stem-overhang mimic. *J. Am. Chem. Soc.* **143**, 11836–11842 (2021).

23. S. Kim, M. Todisco, A. Radakovic, J. W. Szostak, Stereoselectivity of aminoacyl-RNA loop-closing ligation. *J. Am. Chem. Soc.* **147**, 19539–19546 (2025).

24. Y.-L. Li, K. O. Konhauser, M. Zhai, The formation of magnetite in the early Archean oceans. *Earth Planet. Sci. Lett.* **466**, 103–114 (2017).

25. M. Usman, J. Byrne, A. Chaudhary, S. Orsetti, K. Hanna, C. Ruby, A. Kappler, S. Haderlein, Magnetite and green rust: Synthesis, properties, and environmental applications of mixed-valent iron minerals. *Chem. Rev.* **118**, 3251–3304 (2018).

26. N. Tosca, I. Ahmed, B. Tutolo, A. Ashpitel, J. Hurowitz, Magnetite authigenesis and the warming of early Mars. *Nat. Geosci.* **11**, 635–639 (2018).

27. H. Tabata, Y. Sekine, Y. Kanzaki, S. Sugita, An experimental study of photo-oxidation of Fe(II): Implications for the formation of Fe(III) (hydro)oxides on early Mars and Earth. *Geochim. Cosmochim. Acta* **299**, 35–51 (2021).

28. B. H. Patel, C. Percivalle, D. J. Ritson, C. D. Duffy, J. D. Sutherland, Common origins of RNA, protein and lipid precursors in a cyanosulfidic protometabolism. *Nat. Chem.* **7**, 301–307 (2015).

29. J. Xu, V. Chmela, N. J. Green, D. A. Russell, M. J. Janicki, R. W. Gora, R. Szabla, A. D. Bond, J. D. Sutherland, Selective prebiotic formation of RNA pyrimidine and DNA purine nucleosides. *Nature* **582**, 60–66 (2020).

30. S. Ranjan, Z. R. Todd, P. B. Rimmer, D. D. Sasselov, A. R. Babbin, Nitrogen oxide concentrations in natural waters on early Earth. *Geochem. Geophys. Geosyst.* **20**, 2021–2039 (2019).

31. D. C. Catling, K. J. Zahnle, The Archean atmosphere. *Sci. Adv.* **6**, eaax1420 (2020).

32. K. J. Zahnle, R. Lupu, D. C. Catling, N. Wogan, Creation and evolution of impact-generated reduced atmospheres of early Earth. *Planet. Sci. J.* **1**, 11 (2020).

33. N. F. Wogan, D. C. Catling, K. J. Zahnle, R. Lupu, Origin-of-life molecules in the atmosphere after big impacts on the early Earth. *Planet. Sci. J.* **4**, 169 (2023).

34. D. J. Dunlop, O. Ozdemir, *Rock Magnetism: Fundamentals and Frontiers* (Cambridge Univ. Press, 2010).

35. M. R. Safari, F. Matthes, V. Caciuc, N. Atodiresei, C. M. Schneider, K.-H. Ernst, D. E. Burgler, Enantioselective adsorption on magnetic surfaces. *Adv. Mater.* **36**, e2308666 (2024).

36. L. Nagy, W. Williams, A. R. Muxworthy, K. Fabian, T. P. Almeida, P. O. Conbhuı, V. P. Shcherbakov, Stability of equidimensional pseudo-single-domain magnetite over billion-year timescales. *Proc. Natl. Acad. Sci. U.S.A.* **114**, 10356–10360 (2017).

37. N. J. Green, J. Xu, J. D. Sutherland, Illuminating life's origins: UV photochemistry in abiotic synthesis of biomolecules. *J. Am. Chem. Soc.* **143**, 7219–7236 (2021).

38. A. P. Roberts, D. Heslop, X. Zhao, C. R. Pike, Understanding fine magnetic particle systems through use of first-order reversal curve diagrams. *Rev. Geophys.* **52**, 557–602 (2014).

39. R. Day, M. Fuller, V. A. Schmidt, Hysteresis properties of titanomagnetites: Grain-size and compositional dependence. *Phys. Earth Planet. In.* **13**, 260–267 (1977).

40. L. Nagy, R. Moreno, A. R. Muxworthy, W. Williams, G. A. Paterson, L. Tauxe, M. A. Valdez-Grijalva, Micromagnetic determination of the forc response of paleomagnetically significant magnetite assemblages. *Geochem. Geophys. Geosyst.* **25**, e2024GC011465 (2024).

41. K. Ge, W. Williams, L. Nagy, L. Tauxe, Models of maghematization: Observational evidence in support of a magnetic unstable zone. *Geochem. Geophys. Geosyst.* **22**, e2020GC009504 (2021).

42. I. Lascu, J. F. Einsle, M. R. Ball, R. J. Harrison, The vortex state in geologic materials: A micromagnetic perspective. *J. Geophys. Res. Solid Earth* **123**, 7285–7304 (2018).

43. R. J. Harrison, I. Lascu, FORCulator: A micromagnetic tool for simulating first-order reversal curve diagrams. *Geochem. Geophys. Geosyst.* **15**, 4671–4691 (2014).

44. A. P. Roberts, T. P. Almeida, N. S. Church, R. J. Harrison, D. Heslop, Y. Li, A. R. Muxworthy, W. Williams, X. Zhao, Resolving the origin of pseudo-single domain magnetic behavior. *J. Geophys. Res. Solid Earth* **122**, 9534–9558 (2017).

45. L. Nagy, W. Williams, L. Tauxe, A. R. Muxworthy, From nano to micro: Evolution of magnetic domain structures in multidomain magnetite. *Geochem. Geophys. Geosyst.* **20**, 2907–2918 (2019).

46. R. F. Butler, S. K. Banerjee, Theoretical single-domain grain size range in magnetite and titanomagnetite. *J. Geophys. Res.* **80**, 4049–4058 (1975).

47. A. R. Muxworthy, W. Williams, Critical superparamagnetic/single-domain grain sizes in interacting magnetite particles: Implications for magnetosome crystals. *J. R. Soc. Interface* **6**, 1207–1212 (2009).

48. E. S. Nikolaisen, R. Harrison, K. Fabian, S. A. McEnroe, Hysteresis of natural magnetite ensembles: Micromagnetics of silicate-hosted magnetite inclusions based on focused-ion-beam nanotomography. *Geochem. Geophys. Geosyst.* **21**, e2020GC009389 (2020).

49. P. O. Conbhuı, W. Williams, K. Fabian, P. Ridley, L. Nagy, A. R. Muxworthy, MERRILL: Micromagnetic earth related robust interpreted language laboratory. *Geochem. Geophys. Geosyst.* **19**, 1080–1106 (2018).

50. M. R. Safari, F. Matthes, K.-H. Ernst, D. E. Burgler, C. M. Schneider, Enantioselective adsorption on magnetic surfaces. arXiv:2211.12976 [cond-mat.mtrl-sci] (2022).

51. A. Ziv, A. Saha, H. Alpern, N. Sukenik, L. T. Baczewski, S. Yochelis, M. Reches, Y. Paltiel, AFM-based spin-exchange microscopy using chiral molecules. *Adv. Mater.* **31**, e1904206 (2019).

52. J. Gattacceca, P. Rochette, R. B. Scorzelli, P. Munayco, C. Agee, Y. Quesnel, C. Cournede, J. Geissman, Martian meteorites and Martian magnetic anomalies: A new perspective from NWA 7034. *Geophys. Res. Lett.* **41**, 4859–4864 (2014).

53. Z. Cao, Q. Liu, B. P. Weiss, B. Tian, J. Liu, X. Zhang, X. Yu, H. He, Y. Li, H. Tang, Z. Guo, L. Guo, J. Chen, Z. Jiang, X. Zhao, Y. Pan, Submicroscopic magnetite may be ubiquitous in the lunar regolith of the high-Ti region. *Sci. Adv.* **10**, eadn2301 (2024).

54. O. Ben Dor, S. Yochelis, S. Mathew, Y. Paltiel, R. Naaman, A chiral-based magnetic memory device without a permanent magnet. *Nat. Commun.* **4**, 2256 (2013).

55. I. B. Butler, M. A. Schoonen, D. T. Rickard, Removal of dissolved oxygen from water: A comparison of four common techniques. *Talanta* **41**, 211–215 (1994).

56. R. J. Harrison, J. M. Feinberg, FORCinel: An improved algorithm for calculating first-order reversal curve distributions using locally weighted regression smoothing. *Geochem. Geophys. Geosyst.* **9**, Q05016 (2008).

57. R. Egli, VARIFORC: An optimized protocol for calculating non-regular first-order reversal curve (FORC) diagrams. *Global Planet. Change* **110**, 302–320 (2013).

58. F. de la Pena, E. Prestat, J. Lahnemann, V. T. Fauske, P. Burdet, P. Jokubauskas, T. Furnival, C. Francis, M. Nord, T. Ostasevicius, K. E. MacArthur, D. N. Johnstone, M. Sarahan, J. Taillon, T. Aarholt, P. Quinn, V. Migunov, A. Eljarrat, J. Caron, T. Nemoto, T. Poon, S. Mazzucco, S. Borg, N. Tappy, N. Cautaerts, S. Somnath, T. Slater, M. Walls, pietsjoh, *hyperspy/hyperspy: v2.3.0*, version v2.3.0 (2025).

59. C. A. Schneider, W. S. Rasband, K. W. Eliceiri, NIH Image to ImageJ: 25 years of image analysis. *Nat. Methods* **9**, 671–675 (2012).

60. D. Johnstone, P. Crout, C. Francis, M. Nord, J. Laulainen, S. Høgas, E. Opheim, E. Prestat, B. Martineau, T. Bergh, V. J. Femoen, N. Cautaerts, H. W. °Anes, S. Smeets, A. Ross, J. Broussard, S. Dagenborg, S. Huang, S. Collins, P. Vacek, T. Furnival, D. Jannis, I. Hjorth, E. Jacobsen, M. Danaie, A. Herzing, T. Poon, R. Bjørge, A. Iqbal, J. Morzy, T. Doherty, T.

Ostasevicius, T. I. Thorsen, M. von Lany, R. Tovey, P. Vacek, *pyxem/pyxem: v0.20.0*, version v0.20.0 (2024).

61. W. F. Brown, *Micromagnetics* (Interscience, 1963), vol. 18.

62. K. Ray, S. P. Ananthavel, D. H. Waldeck, R. Naaman, Asymmetric scattering of polarized electrons by organized organic films of chiral molecules. *Science* **283**, 814–816 (1999).

63. S. Buessecker, H. Imanaka, T. Ely, M. L. Wong, J. P. Harrison, A. A. Pavlov, M. W. Claire, M. G. Trainer, K. J. Zahnle, R. E. Summons, N. J. Tosca, Mineral-catalysed formation of marine NO and N_2O on the anoxic early Earth. *Nat. Geosci.* **15**, 1056–1063 (2022).

64. W. Williams, R. Moreno, A. R. Muxworthy, G. A. Paterson, L. Nagy, L. Tauxe, U. Donardelli Bellon, A. A. Cowan, I. Ferreira, Vortex magnetic domain state behavior in the day plot. *Geochem. Geophys. Geosyst.* **25**, e2024GC011462 (2024).

65. R. Naaman, D. H. Waldeck, Chiral-induced spin selectivity effect. *J. Phys. Chem. Lett.* **3**, 2178–2187 (2012).
